# The use of localised CBCT to image inflammatory collateral cysts: a retrospective case series demonstrating clinical and radiographic features

**DOI:** 10.1007/s40368-019-00488-8

**Published:** 2019-11-15

**Authors:** M. Dave, F. Thomson, S. Barry, K. Horner, N. Thakker, H. J. Petersen

**Affiliations:** 1 Division of Dentistry, Faculty of Biology, Medicine and Health, Manchester Academic Health Science Centre, School of Medical Sciences, The University of Manchester, Oxford Road, Manchester, M13 9PL UK; 2 University Dental Hospital of Manchester, Higher Cambridge Street, Manchester, M15 6FH UK

**Keywords:** Radiology, Cysts, Oral, Surgery, Pathology

## Abstract

**Introduction:**

Inflammatory collateral cysts are uncommon cysts primarily affecting first permanent molars during their eruption. There are diagnostic challenges that can be overcome with CBCT imaging. However, given the paediatric age group for this condition, there are patient cooperation and radiation dose factors to consider when justifying the scan. The aim of this case series study is to illustrate the value of CBCT in imaging and diagnosing inflammatory collateral cysts in paediatric patients, to highlight the need for a multidisciplinary approach for this uncommon pathological condition and to review the relevant literature.

**Case series description and results:**

We present three patients aged between 6 and 11 years of age with inflammatory collateral cysts affecting their first or second permanent molars for which CBCT imaging was utilised. All patients underwent cyst enucleation with preservation or extraction of affected teeth under general anaesthesia.

**Discussion:**

Inflammatory collateral cysts are likely to be under reported given their indistinct clinical features and radiological signs. Conventional planar radiographs may not reveal this lesions size and full extent. CBCT overcomes these limitations; however, careful assessment of patient cooperation is needed and a low-dose protocol should be used.

**Conclusions:**

CBCT can provide useful imaging information which is difficult to obtain using conventional radiography, especially in cases where an inflammatory collateral cyst is suspected.

## Introduction

The inflammatory collateral cyst is recognised in the World Health Organisation classification of head and neck tumours as primarily affecting mandibular permanent molars during their eruption (Carlson 2007; Friedrich et al. 2014; Thikkurissy et al. 2010). It is uncommon with a reported 1.6–4.5% population prevalence on third molars and 3–5% prevalence on first and second molars (Mufeed et al. 2009; Philipsen et al. 2004). The aetiology is unclear and other names have been used for it including paradental cyst and buccal bifurcation cyst. Clinical presentation is usually of a progressively expanding swelling buccal to a developing permanent molar causing bone resorption and altering the crown’s angulation. Symptoms are normally evident once the lesion has sufficiently advanced and has become acutely inflamed through an infectious process (Ramos et al. 2012).

As the inflammatory collateral cyst is commonly situated on the buccal surface, conventional dental radiography will result in a radiolucency superimposed over the roots of the affected tooth with a lamina dura of normal appearance (Philipsen et al. 2004). Radiological interpretation using conventional radiographs can be challenging and, with non-specific clinical signs, there is the possibility of a misdiagnosis and ineffective treatment planning. Cross-sectional imaging with cone beam computed tomography (CBCT) would overcome these limitations; however, the clinical dilemma is that most patients are aged between 6 and 11 years (Thikkurissy et al. 2010). Patient cooperation, radiation exposure and the diagnostic value of further imaging need careful consideration.

The aim of this case series is to illustrate the value of CBCT in imaging and diagnosing inflammatory collateral cysts in paediatric patients, to highlight the need for a multidisciplinary approach for this uncommon pathological condition and to review the relevant literature. All patients were medically unremarkable and referred by their General Dental Practitioners to the Paediatric Department, University Dental Hospital of Manchester (UDHM) for further management (summarised in Table 1). As per local policy, all CBCT scans were performed using a paediatric low-dose protocol (Hidalgo Rivas et al. 2015). Written consent for publication was obtained from parents/guardians with parental responsibility.

**Table 1:** Summary of the patients presented in this case series

Case number	Case characteristics	Diagnostics	Treatment plan	Results	Follow-up
1	8-year-old girl with pain and swelling associated with her LR6	Clinical examination revealed buccal expansion and the LR6 was negative to sensibility testing A PR was taken which confirmed a furcal radiolucency A true occlusal radiograph was attempted; however, it did not provide sufficient coverage Further imaging with a CBCT scan was undertaken	Extraction of the LR6 under general anaesthetic and enucleation of the cyst	Histopathology confirmed an inflammatory odontogenic cyst consistent with the clinical diagnosis of an inflammatory collateral cyst	6-month clinical and radiographic follow-up showed complete resolution of the cyst
2	11-year-old boy referred for management of molar-incisor hypomineralisation affecting all first permanent molars	Clinical examination revealed post-eruptive breakdown and large composite restorations associated with his first permanent molars. All first permanent molars were positive to sensibility testing A PR revealed an incidental radiolucency associated with the LL6 and a suspicious furcal radiolucency associated with the LR6. Further imaging with a CBCT scan was undertaken	Enucleation of the cyst under general anaesthetic with preservation of the LL6. A second pre-operative PR was planned nearer the time of surgery	12 months after the initial examination, there was buccal expansion associated with the LR6 and a radiolucency associated with its bifurcation. Both cysts were enucleated under general anaesthetic (and both associated first permanent molars were retained). A diagnosis of bilateral inflammatory collateral cysts were made, as confirmed through histopathology	6-month clinical and radiographic follow-up showed complete resolution of both cysts
3	6-year-old girl with pain and facial swelling associated with her LL6	Clinical examination revealed buccal expansion associated with the LL6. The tooth was positive to sensibility testing A PR confirmed a furcal radiolucency associated with the LR6 and LL6. Further imaging with a CBCT scan was undertaken	Enucleation of LR6 and LL6 cysts under general anaesthetic with preservation of both teeth	Histopathology confirmed an inflammatory odontogenic cyst consistent with the clinical diagnosis of bilateral inflammatory collateral cysts	6-month clinical and radiographic follow-up showed complete resolution of both cysts

## Case series description and results

### Case 1

An eight-year-old girl presented to her primary care General Dental Practitioner with pain from her lower right first permanent mandibular molar (LR6). She reported two previous episodes of pain that had resolved spontaneously; however, this instance kept her awake at night and was accompanied by an acute facial swelling spreading to the right cheek. The patient was prescribed a course of metronidazole and referred to the UDHM.

**Fig. 1:**
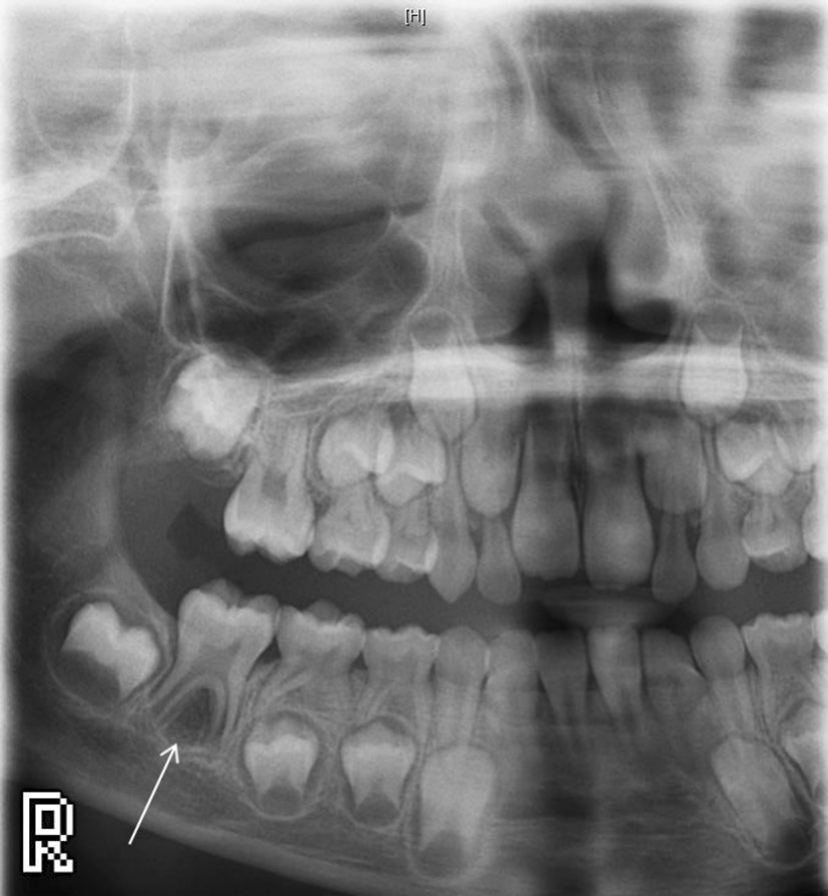
Unilateral panoramic radiograph showing a furcal radiolucency associated with the lower right first mandibular molar

Examination revealed buccal expansion localised to the LR6 and the tooth was reliably negative to sensibility testing. There were no abnormalities of note on any other first permanent molars. A unilateral panoramic radiograph (PR) revealed a LR6 furcal radiolucency extending over the apices with loss of lamina dura (Fig. 1). There was no caries or other apparent dental pathosis, with the lesion being suggestive of either periodontal bone loss or cystic change.

**Fig. 2:**
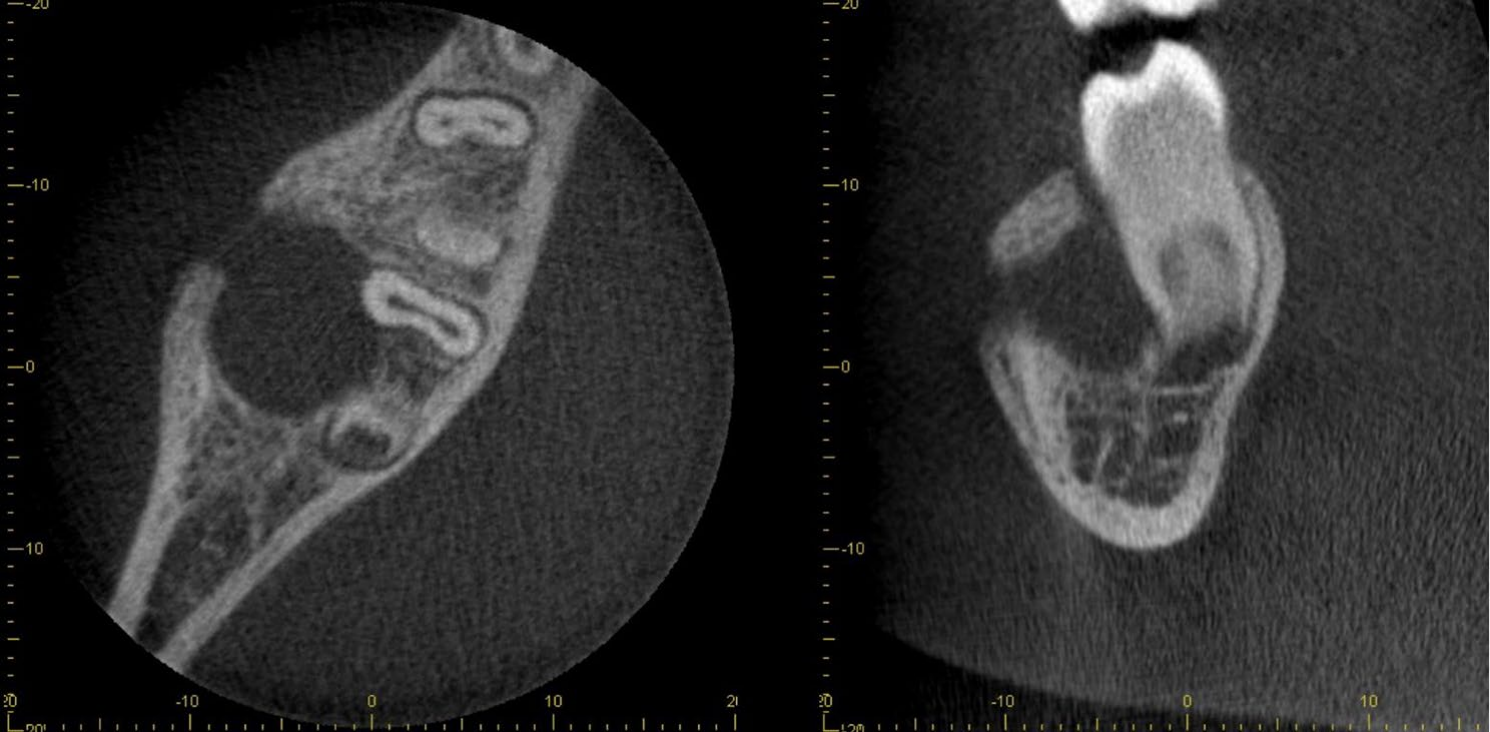
CBCT showing a well-defined buccal cyst involving the lower right first mandibular molar with buccal expansion and perforation of the expanded buccal cortex. It is of note that the lesion is tilting the roots lingually and subsequently the crown buccally

A true occlusal radiograph was attempted but gave inadequate coverage and a repeat image was not exposed because it was judged unlikely to be improved. A localised CBCT scan was performed which showed a well-defined spherical radiolucency centred over the furcation of the LR6. There was loss of lamina dura with a radiolucent lesion of an extensive size (11.5 × 11.5 × 10.5 mm) with buccal expansion, representing an appearance consistent with a chronically infected inflammatory collateral cyst (Fig. 2).

The patient was referred for extraction of the LR6 under general anaesthesia with the histopathology report confirming an inflammatory odontogenic cyst consistent with the clinical diagnosis of an inflammatory collateral cyst. Clinical and radiographic examination at the 6-month post-operative review confirmed complete resolution of the cyst.

### Case 2

An eleven-year-old boy was referred by his General Dental Practitioner regarding management of his broken down first permanent molars. Clinical and radiographic investigations revealed a diagnosis of molar-incisor hypomineralisation with post-eruptive breakdown of both the upper left and lower left first permanent molars and large composite restorations on the contralateral side. All first permanent molars were reliably positive to sensibility testing with no buccal expansion.

**Fig. 3:**
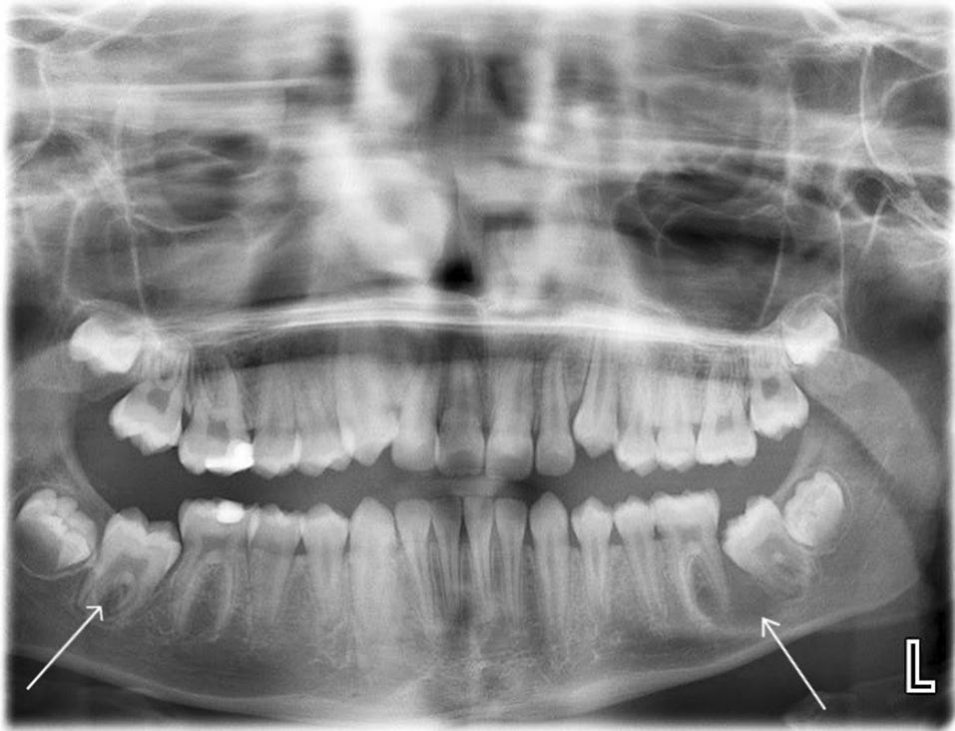
Panoramic radiograph showing a radiolucency extending between the lower left first and second permanent molars. Also note the radiolucent bifurcation of the unerupted lower right second permanent molar

A PR revealed an incidental radiolucency in the lower left first and second permanent molar region suggestive of a cyst. In addition, there was a radiolucency situated on the unerupted lower right second permanent molar furcation (Fig. 3). A localised CBCT scan was performed of the lower left first permanent molar region only as it was not deemed justifiable to expose the contralateral side given the absence of any signs and symptoms and indistinct radiological features.

**Fig. 4:**
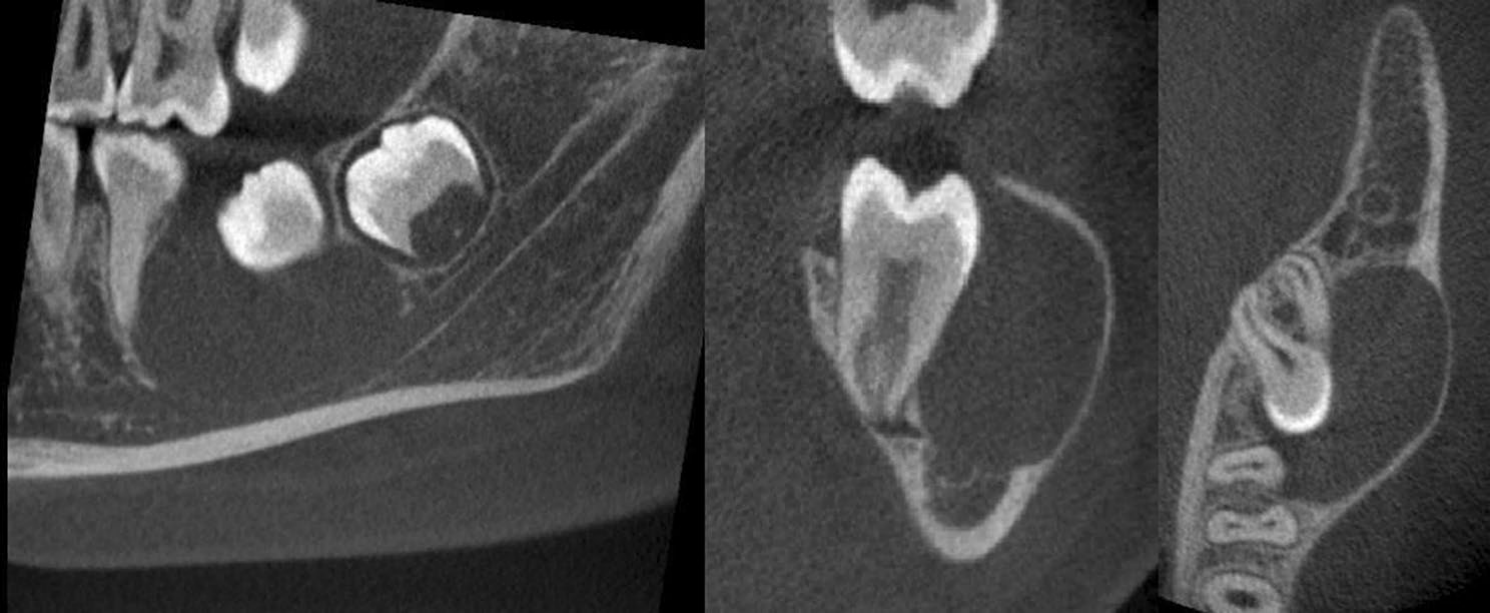
The CBCT shows a corticated monolocular radiolucency associated with the lower left mandibular first permanent molar. There is buccal cortex expansion nonetheless, the superior border of the inferior alveolar nerve does not show any signs of erosion

The CBCT showed features of a well-corticated, unilocular radiolucency consistent with the appearance of an inflammatory collateral cyst (Fig. 4). Whilst the cyst was situated buccal to lower left second permanent molar, it was associated with the lower left first permanent molar.

The patient and his parents were informed of the guarded long-term prognosis for all first permanent molars. Enucleation of the cyst was planned under general anaesthetic and, with a waiting list of approximately 12 months; a clinical and radiographic examination was scheduled prior to the surgery to assess any further cystic growth and changes on the lower right first permanent molar region. A second PR closer to the time of surgery was considered to be justified in view of the suspicion about the contralateral side, especially with evidence from literature showing 23.6–37.5% of patients having bilateral lesions (Philipsen et al. 2004).

**Fig. 5:**
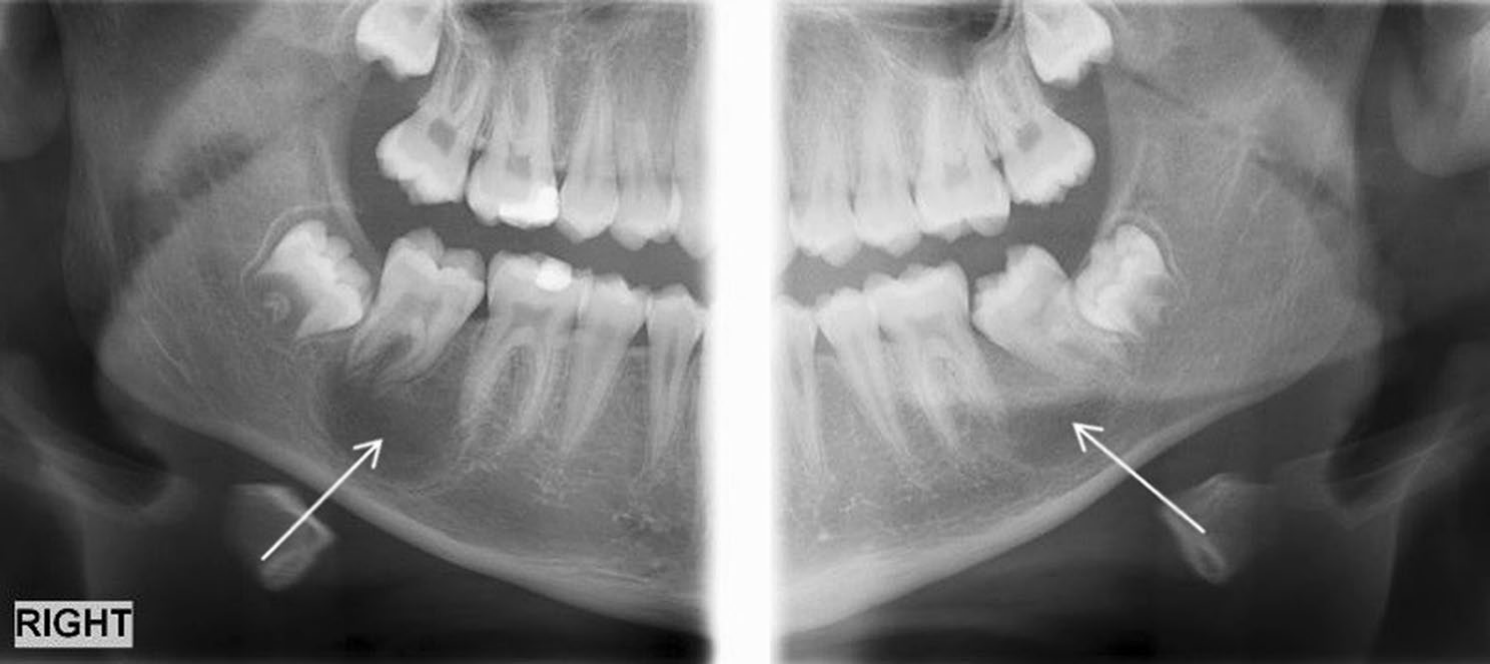
Panoramic radiograph taken prior to surgery showing well-defined radiolucencies associated with both the lower left first permanent molar and lower right second permanent molar

**Fig. 6:**
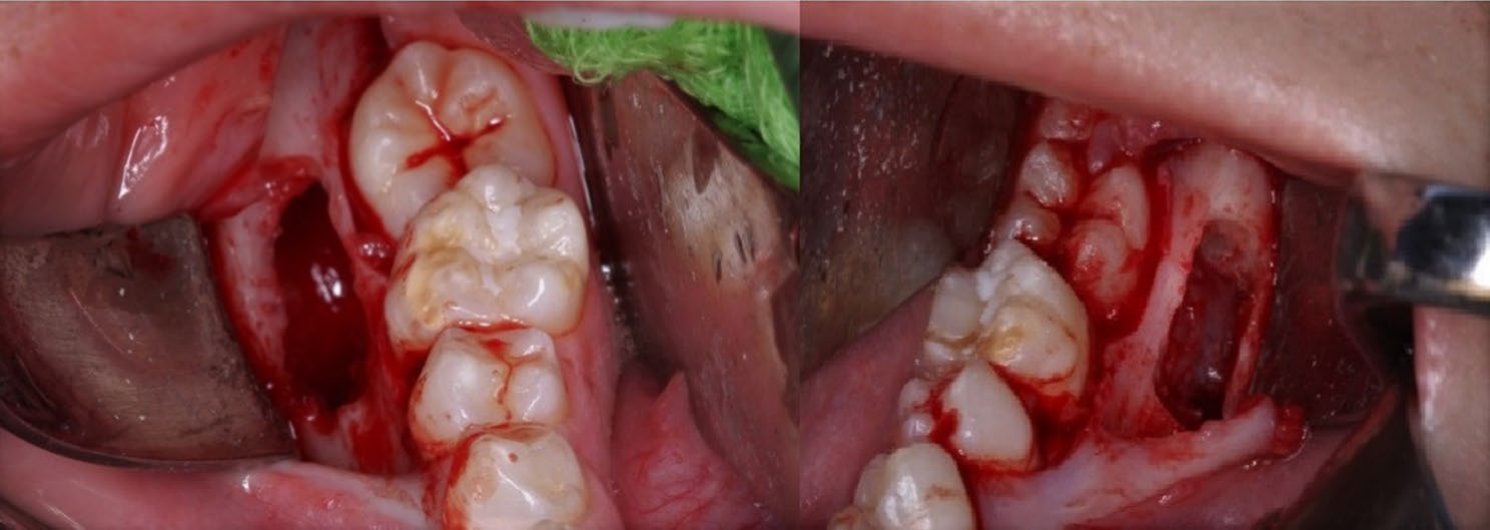
Peri-operative photograph showing cyst enucleation with preservation of their associated teeth

**Fig. 7:**
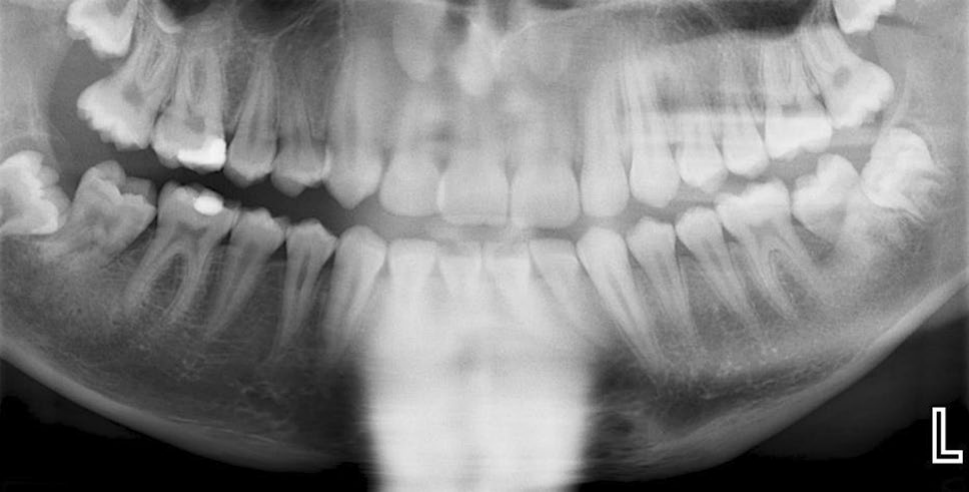
The 6-month post-operative review confirmed resolution of radiolucencies associated with both the lower left first permanent molar and lower right second permanent molar

Twelve months after the initial examination, there was palpable buccal expansion and the lower right second permanent molar had developed a well-corticated radiolucency (Fig. 5). The tooth had sufficiently erupted and sensibility testing revealed a positive response. A further CBCT scan was not taken as it would not change management. A provisional diagnosis of bilateral inflammatory collateral cysts was made and surgical enucleation of both cysts completed under general anaesthesia (Fig. 6). Histopathology, in conjunction with clinical findings, confirmed the diagnosis of bilateral inflammatory collateral cysts. Clinical and radiographic examination at the 6-month post-operative review showed no signs of inflammation and resolution of the radiolucencies associated with both mandibular first permanent molars (Fig. 7).

### Case 3

**Fig. 8:**
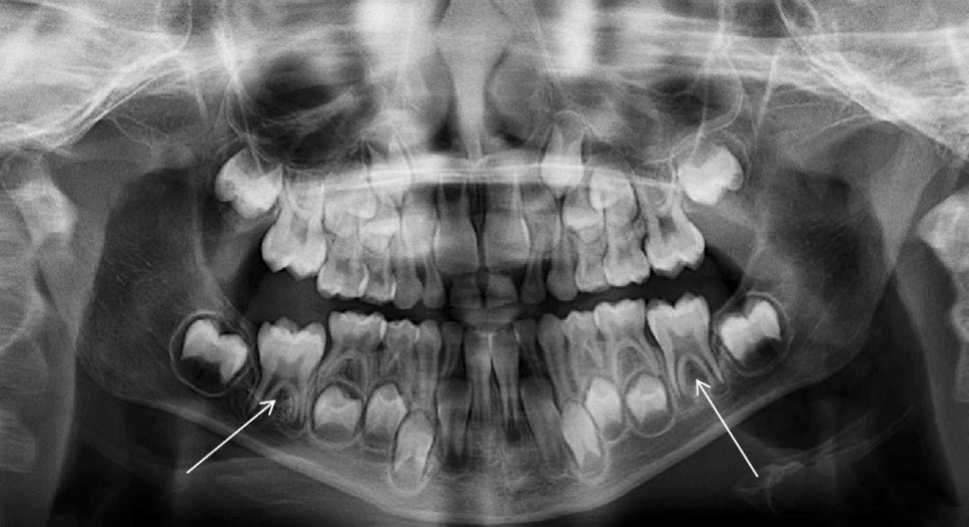
Panoramic radiograph showing radiolucencies associated with the bifurcation of the lower left and right first permanent molars

**Fig. 9:**
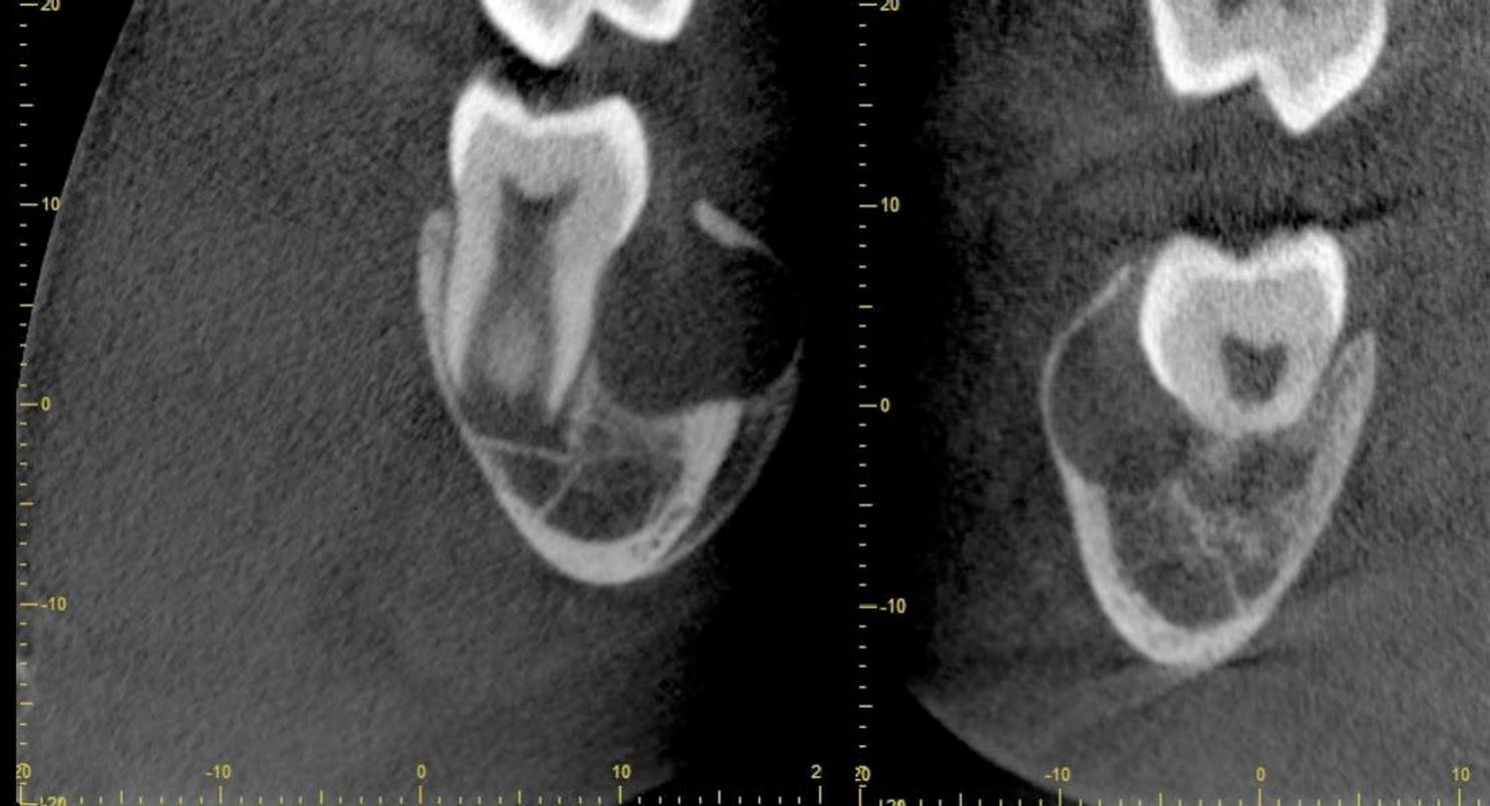
The CBCT scan revealed a well-defined, spherical radiolucency situated buccal to both the lower right and lower left first molar regions overlying the furcation with associated thinning and perforation of the buccal cortex

**Fig. 10:**
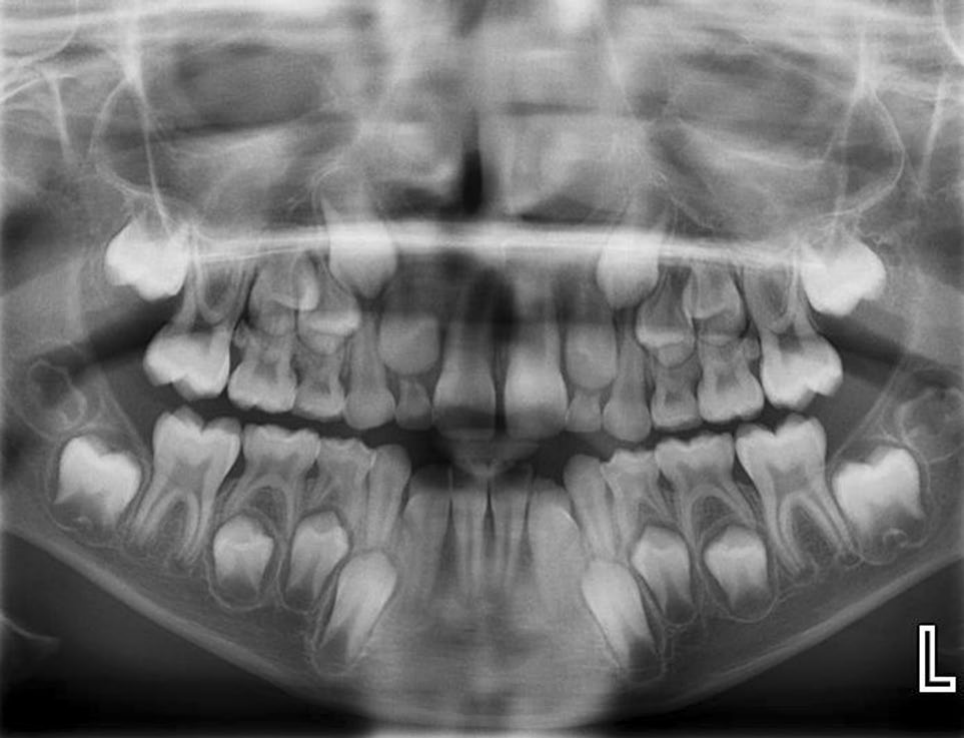
The 6-month post-operative review confirmed resolution of radiolucencies associated with both mandibular first permanent molars

A six-year-old girl presented with a history of three previous episodes of pain associated with a persistent left-sided facial swelling that had been unsuccessfully managed with oral antibiotics by her General Dental Practitioner. On examination, the lower left first permanent molar was partially erupted with buccal expansion and tenderness. It was positive to sensibility testing and a PR (Fig. 8) confirmed a radiolucency associated with its bifurcation and also a smaller radiolucency in the lower right first permanent molar bifurcation. A CBCT scan was performed which confirmed inflammatory collateral cysts associated with both mandibular first permanent molars (Fig. 9). Additionally, there was a florid periosteal reaction on the left mandible correlating with the history of long-term swelling. Both cysts were enucleated under general anaesthesia with histopathology confirming bilateral inflammatory odontogenic cysts consistent with the clinical diagnosis of bilateral inflammatory collateral cysts. At the 6-month post-operative review, there were no signs of inflammation and radiographically, there was resolution of both inflammatory collateral cysts (Fig. 10).

All patients presented were followed up for 6 months clinically and radiographically with a PR. There was complete resolution of the cysts and changes in the angulation of associated teeth to their correct position within the line of the arch. All patients were discharged back to the care of their dentists.

## Discussion

The 4th edition of the World Health Organisation (WHO) Classification of Head and Neck tumours: Odontogenic and Maxillofacial Bone Tumours reintroduced cysts into the classification (El-Naggar et al. 2017; Speight and Takata 2018). Two groups of cysts have been detailed: odontogenic and non-odontogenic developmental cysts, and odontogenic cysts of inflammatory origin with two constituent subgroups in the latter, the radicular and inflammatory collateral cyst (Carlson 2007).

### Inflammatory collateral cyst

Inflammatory collateral cysts have been previously referred to as buccal bifurcation and paradental cysts in literature, along with many other synonyms (Table 2) (Wright and Vered 2017). The term buccal bifurcation cyst describes cysts situated buccal to first or second permanent molars, often during eruption in children of respective dental age (David et al. 1998; Friedrich et al. 2014; Ramos et al. 2012). Cysts in the same position situated on third permanent molars have been described as paradental cysts; however, these terms have been used interchangeably (Thikkurissy et al. 2010). Both cysts are inflammatory collateral cysts and should be referred as such, as defined by the WHO classification and as acknowledged in this case series (El-Naggar et al. 2017).

**Table 2:** Synonyms for the inflammatory collateral cyst (Corona-Rodriguez et al. 2011; Mufeed et al. 2009; Philipsen et al. 2004; Ramos et al. 2012; Silva et al. 2003)

Inflammatory collateral cyst	
Buccal bifurcation cyst	Paradental cyst
Mandibular infected buccal cyst	Mandibular infected buccal cyst
Juvenile paradental cyst	Craig’s cyst
Circumferential dentigerous cyst	Eruption pocket cyst
Paradental cyst	Inflammatory paradental cyst

Inflammatory collateral cysts develop during the tooth’s eruption, which is reflected by their average age of presentation being 6–11 years. Those affecting third molars tend to present in the third decade of life due to late eruption and high tendency for impaction (Jones et al. 2006; Thikkurissy et al. 2010). Bilateral inflammatory collateral cysts have also been reported in literature, with the patient reported in ‘case 2’ an example of bilateral cyst presentation (Corona-Rodriguez et al. 2011; Ramos et al. 2012).

The pathogenesis of inflammatory collateral cysts is postulated to be a combination of the accumulation of microbial plaque and a break in oral mucosa during tooth eruption. This initiates the release of inflammatory mediators and the formation of non-keratinised, hyperplastic stratified squamous cystic lining with inflammatory infiltration on the lateral root surface (De Grauwe et al. 2018; Maruyama et al. 2015; Philipsen et al. 2004). Factors that predispose to plaque accumulation such as enamel pearls at the bifurcation are likely to be contributing factors. It is rare for inflammatory collateral cysts to affect tooth vitality; hence, they can progress asymptomatically until an infectious process initiates swelling and pain, or until they are detected as incidental findings (Oenning et al. 2018; Ramos et al. 2012). The symptoms of pain, discomfort and trismus are mainly due to inflammation of the pericoronal tissue, especially on mandibular third molars (Chrcanovic et al. 2011; El-Naggar et al. 2017; Lo Muzio et al. 2017). Whilst this cyst predominantly affects molars, there have been reports of it affecting other teeth such as mandibular premolars (Shear and Speight 2008).

An initial clinical examination should be undertaken to examine the appearance of the local site and overlying skin, status of regional lymph nodes and to ascertain the extent of any bony expansion. In addition, patient compliance and the prognosis of the associated tooth should form part of the assessment. Several features of the inflammatory collateral cyst are presented in Table 3 (Jones et al. 2006). A biopsy can confirm the diagnosis of an odontogenic inflammatory cyst; however, categorisation using the WHO classification (4th edition) depends on further clinical and radiological features (Silva et al. 2003).

**Table 3:** Clinical, radiographic and histological features of an ICC (Corona-Rodriguez et al. 2011; Lizio et al. 2011; Ramos et al. 2012; Silva et al. 2003)

Clinical features	Radiographic features	Histological features
Rotation of an erupting mandibular molar	Intact lamina dura and normal periodontal ligament space	Fibrous capsule—continuous with the pericoronal tissue or cemento-enamel junction
Buccal expansion	‘U shaped’ radiolucency superimposed over roots at the bifurcation	Cholesterol clefts, foamy macrophages and inflammatory infiltration
Canting of the occlusal plane towards the buccal surface	Smaller apices and prominent buccal cusps	Hyperplastic stratified squamous epithelium
Vital tooth		

### Conventional and cross-sectional radiography

Initial radiographic examination should be undertaken with conventional dental views such as periapical radiographs or PRs showing the entirety of the affected tooth and the entire boundaries of the lesion. It is noteworthy that bilateral cases are frequent enough to consider imaging the contralateral side. Careful examination of the lamina dura should be undertaken as loss of lamina dura is likely to indicate other dental pathosis such as periodontitis (Ramos et al. 2012). The inflammatory collateral cyst is situated buccal to the affected tooth, causing a superimposed radiolucency over the roots. This appearance can be diagnostically challenging to distinguish from a radicular or lateral periodontal cyst (Philipsen et al. 2004). A true occlusal radiograph can be useful to determine bony expansion; however, cooperation, especially in the young patient, can prove challenging. Radiographic aberrations can be difficult to correctly identify and delineate from normal development or other dental pathosis; hence, it is imperative to seek further advice from a specialist Dental and Maxillofacial Radiologist before considering interventional treatment if deemed necessary (Dave and Horner 2017).

Cross-sectional imaging with CBCT overcomes the limitations of superimposition as with planar radiography. However, it delivers a higher dose of radiation compared to conventional techniques; hence, the latter should always be attempted first (White et al. 2014). Clinicians should aim to keep the radiation dose as low as reasonably practicable which is particularly important in children who are at a greater risk of stochastic damage compared to adults for a given dose of radiation (Brenner and Hall 2007). The ‘Image Gently in Dentistry’ campaign outlines methods of reducing radiation exposure (White et al. 2014). An optimised low-dose paediatric protocol should be used if feasible and the patient carefully judged for suitable cooperation because of the risk of movement artefact. The tube current and exposure time are reciprocal factors in X-ray exposure; so, if both can be adjusted, a short exposure time/higher current combination will limit the impact of any patient movement. It is of note that the use of magnetic resonance imaging (MRI) is likely to produce greater diagnostic yield on imaging the cyst and its relation to anatomical structures without any exposure to ionising radiation. Nonetheless, many limitations have hindered its progress in dentistry, including artefacts from dental restorations and orthodontic brackets (Deana and Alves 2017). Furthermore, patient cooperation when undergoing MRI may be challenging, particularly in children.

The treatment of inflammatory collateral cysts involves enucleation and extraction or preservation of the affected teeth (Ramos et al. 2012). Nonetheless, confirmation of diagnosis through histopathological examination is necessary, as there have been cases where other lesions have mimicked the appearance of an inflammatory collateral cyst such as odontogenic keratocysts (Borgonovo et al. 2014).

## Conclusion

It is hoped that further awareness about inflammatory collateral cysts will encourage clinicians to be more vigilant especially when examining radiographs that show the furcation of developing molars. This case series has exemplified that radiolucencies associated with molars do not always indicate loss of vitality and subsequent need for root canal treatment or extraction. Radiographic examination should always be preceded by a full clinical examination, including sensibility testing and referral to a specialist if required. CBCT can provide imaging information which is difficult to obtain using conventional radiography in cases where an inflammatory collateral cyst is suspected.
